# Mixed Metal Oxide by Calcination of Layered Double Hydroxide: Parameters Affecting Specific Surface Area

**DOI:** 10.3390/nano11051153

**Published:** 2021-04-28

**Authors:** Su-Bin Lee, Eun-Hye Ko, Joo Y. Park, Jae-Min Oh

**Affiliations:** 1 Department of Energy and Materials Engineering, Dongguk University-Seoul, Seoul 04620, Korea; sban0103@naver.com (S.-B.L.); gksmf__2@naver.com (E.-H.K.); 2 Discipline of Information Technology, Media and Communication, Murdoch University, Western Australia 6150, Australia

**Keywords:** mixed metal oxide, layered double hydroxide, calcination, specific surface area, metal composition

## Abstract

Mixed metal oxide (MMO) is one of the widely utilized ceramic materials in various industries. In order to obtain high performance, the specific surface area of MMO should be controlled. Calcination of layered double hydroxide (LDH) is a versatile way to prepare MMO with homogeneous metal distribution and well-developed porosity. Although researchers found that the specific surface area of LDH-originated MMO was relatively high, it had not been systematically investigated how the surface area is controlled under a certain parameter. In this review, we summarized LDH-originated MMO with various starting composition, calcination temperature, and pore developing agent in terms of specific surface area and porosity. Briefly, it was represented that MMOs with Mg-Al components generally had higher specific surface area than Mg-Fe or Zn-Al components. Calcination temperature in the range 300–600 °C resulted in the high specific surface area, while upper or lower temperature reduced the values. Pore developing agent did not result in dramatic increase in MMO; however, the pore size distribution became narrower in the presence of pore developing agents.

## 1. Introduction

Mixed metal oxides (MMO) are utilized in various application fields including catalysts, electrodes, adsorbents, etc. In the technical point of view, they can be prepared to have wide variety of composition, crystallinity, particle size, etc. In other word, their electronic structure, surface chemistry, and the degree of unsaturated coordination bonds could be intentionally controlled for the industrially beneficial properties such as adjusted adsorption property and catalytic activity [1,2,3,4,5,6,7,8]. For example, MMOs can be prepared with various metal composition, normally as Mg-Al, Mg-Fe, Zn-Al, etc. The MMO composed of Co^2+^ and Fe^3+^ was known to be effective in low-temperature CO oxidation if it had high specific area, modified surface oxygen, and potential metal-metal interaction [9]. Li et al. reported that ternary MMO having Cu-Zn-Al composition was effective in H_2_S removal when it had high specific surface area and small crystallite size [10].

Reflecting the above-mentioned importance, diverse synthetic routes have been developed such as ceramic method, sol-gel route, hydrothermal treatment, etc. [11,12]. The ceramic method is the most simple and common way to synthesize metal oxide. This involves precursors such as oxides, carbonates, or metal-salts mixed together and heated at a desired temperature to induce diffusion between two components [13]. In spite of high accessibility and easy preparation, this method has several drawbacks. High energy and temperature are required for reaction and multiple steps are needed for the preparation of complicated composition. Sol-gel route is utilized to produce inorganic polymers or ceramics in stoichiometric manner [14]. The synthesis is proceeded through following steps: hydrolysis of precursors to form sol, formation of gel via polycondensation, and aging and drying to form a dense gel. The final product is often dehydrated to obtain intended shape of materials. The MMOs prepared by sol-gel route are known to have high specific surface area with well-developed porosity [15]. Although sol-gel is an economical way to produce a stoichiometric product, it often accompanies shrinkage or crack of product during the drying process, resulting in an unexpected property [16]. Hydrothermal treatment is generally used for substances that are usually insoluble in ordinary temperature and pressure (<100 °C, 1 bar). Mataji et al. [17] prepared Zn-Fe/Zr-MMO in a cost-effective manner utilizing hydrothermal method at 180 °C for 2 h. This method is useful in controlling particle size with high homogeneity; however, its unidentified reaction mechanism hindered minute control of reaction process [18].

Recently, calcination of layered double hydroxide (LDH) has been suggested as an alternative way to prepare MMO. The LDH, also known as anionic clay, is composed of positively charged brucite-like layers and exchangeable anions. Its general chemical formula is described as [M^2+^_1−x_M^3+^_x_(OH)_2_](A^n−^)_x/n_∙mH_2_O, where M^2+^ is a divalent metal cation (e.g., Mg^2+^, Zn^2+^, Ca^2+^, etc.), M^3+^ is a trivalent cation (e.g., Al^3+^, Fe^3+^, Cr^3+^, etc.), and A^n−^ is an exchangeable anion such as CO_3_^2−^, NO_3_^2−^, SO_4_^2−^, etc. Due to the variation in metal composition, even ternary or quaternary metal composition is possible [19,20,21,22,23,24], high anionic exchange capacity, and anisotropic structure, LDHs are widely studied as catalyst, catalytic support, drug delivery carrier, pollutant adsorbent, etc. [25,26,27,28,29,30,31,32]. It is also well-known that LDH, upon calcination, undergoes phase-transformation to MMO through sequential dehydration, dehydroxylation, and gasification of interlayer anions. For example, Mg_6_Al_2_(OH)_16_(CO_3_)∙4H_2_O (hydrotalcite) stoichiometrically alters to Mg_3_AlO_4.5_ (MMO) when they are calcined at an appropriate temperature, between 300 °C and 600 °C. These MMOs have high thermal stability and relatively large specific surface area [33]. Furthermore, chemical properties of LDH-originated MMO can be controlled by differing the metal components and calcination temperature [34,35]. Thus, the MMOs prepared by this method are widely utilized as catalyst, electrochemical capacitor, antibacterial activity, and adsorbent [36,37,38,39,40,41,42,43].

**Scheme 1:**
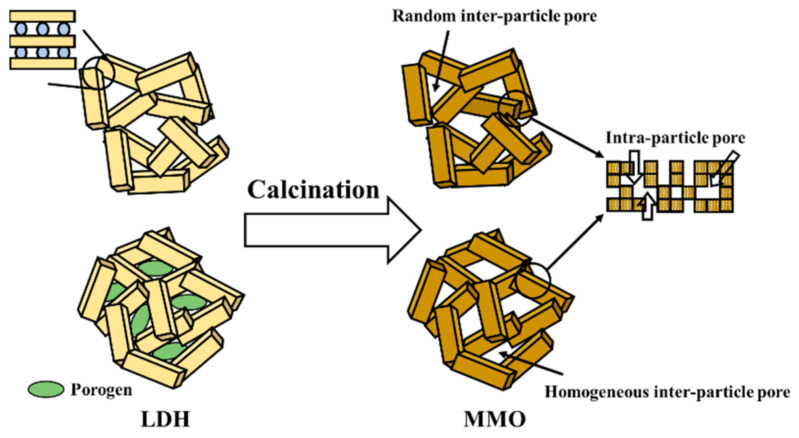
Intracrystalline structure and porosity of MMO obtained from LDH with and without porogen.

One of the characteristic features of MMO derived from LDH is the development of porous structure through gasification process, resulting in relatively high specific surface area. Consequently, LDH-originated MMO have been reported to show high anionic removal capacity [44,45]. The phase transformation mechanism from LDH to MMO is not clearly defined yet; however, it is generally accepted that there evolved interstratified M(II)O domains which are connected to each other by M(III)O tetrahedron. According to the literature survey, we could hypothesize two reasons of porosity in MMO derived from LDH, as illustrated in Scheme 1. First, calcined LDH is used to preserve the morphology of pristine LDH where several M(II)O domains are separated with an appropriate distance. The intraparticle space among M(II)O domains would be the first reason of porosity. Second, the calcination process makes MMO particles form agglomerates, of which the interparticle space provides pores.

Although there has been extensive research on the utility of LDH-originated MMO and most of the studies highlighted the importance of specific surface area, to the best of our knowledge, there have not been integrated approaches to comprehend the parameters that affect the specific surface area of MMO. In this contribution, we surveyed the literature on MMOs prepared by LDH to summarize the parameters affecting specific surface area of LDH-based MMO. First of all, the relationship between metal composition of LDH and the specific surface area of MMO was examined. According to the frequency of report, Mg-Al, Zn-Al, and Mg-Fe composition will be mainly summarized. Then, the effect of calcination temperature parameter on the surface area of MMO was also investigated. In addition, literature on the pore developing template in LDH-originated MMO was also searched. Although the templated method is not an industry friendly method, the pore control by template is important in the technical point of view. By selecting the appropriate metal composition in LDH, controlling calcination temperature, or incorporating pore-developing template in LDH synthesis, it is possible to control the specific surface area and porosity of MMOs.

## 2. Specific Surface Area of MMO Prepared from Calcined LDH

### 2.1. Importance of Specific Surface Area Control in Industiral Application of MMO

#### 2.1.1. Market and Industry Trend of MMO

Metal oxide is one of the inorganic materials widely used as catalysts, electrodes, and adsorbents [46], and is used in all fields such as construction and industry, occupying a very important position in the infrastructure industry. MMOs are also used in various range of applications such as semiconductors, ceramics, and medicine as antifungal agents [47,48]. In particular, advanced ceramics are being used in various applications such as electronics, transportation, medical, environment, defense and security, and chemical industries, and the global market size is expected to reach $10,410 billion by 2021 [49].

In addition, mixed metal oxides have been used in a number of industrially important synthetic conversions used in numerous pharmaceutical and natural products [50]. In the case of the environmental industry, Mochane et al. [11] demonstrated that the use of MMOs spans a wide range of fields, including food packaging, energy, water purification, and gas detection. Environmental ceramics are used in energy and environmental fields through functions such as energy storage, conversion, adsorption, filtration, and decomposition of environmental pollutants [51]. As interest in the global environment increases, demand for ceramic-related materials in the environment sector is expected to increase significantly.

As such, the application of MMO is remarkably growing with the rapid industrial development of the electronical and electronic, information and communications, and automotive industries. In particular, the ceramic material and parts industry is expected to continue to grow due to the rapid development of the new growth engine industry due to the expansion of the customized high functional material market. However, the ceramic industry is the most competitive in the global market and needs to continuously create new markets through the development of products with new functions [52]. Thus, it is required to expand the government’s support for small and medium-sized enterprises, continuous research on MMO-related technologies, and foster professional manpower.

#### 2.1.2. Importance of Controlling the Specific Surface Area in Industrial Application

MMO plays a very important role in industrial research and applications as various types of catalysts due to their high surface area and reactive sites [53]. Notably, some of the MMOs are attracting great attention in industrial applications because they play a high catalyst role in numerous reactions by controlling a high specific surface area. This is because as the specific surface area increases, which adsorbs a wide variety of chemicals, the reaction time may be shortened, and the conversion rate of the reactant may increase rapidly [50]. Mixed metal oxide with a high surface area could replace many heterogeneous catalysts, increasing the efficiency of the application.

### 2.2. Controlling the Specific Surface Area of MMO by Calcining Pure LDH

#### 2.2.1. Metal Composition of LDH

- General features

Among various literature on LDH-originated MMO, the most frequently found composition is Mg-Al. To study metal composition on S_BET_ and porosity, we chose two more compositions, Zn-Al and Mg-Fe, respectively, in which divalent metal and trivalent metal are replaced by Zn and Fe, respectively. For statistical analyses, we searched 50, 50, and 20 papers for MgAl-, ZnAl-, and MgFe-MMO, respectively, and summarized them in Table 1.

**Figure 1:**
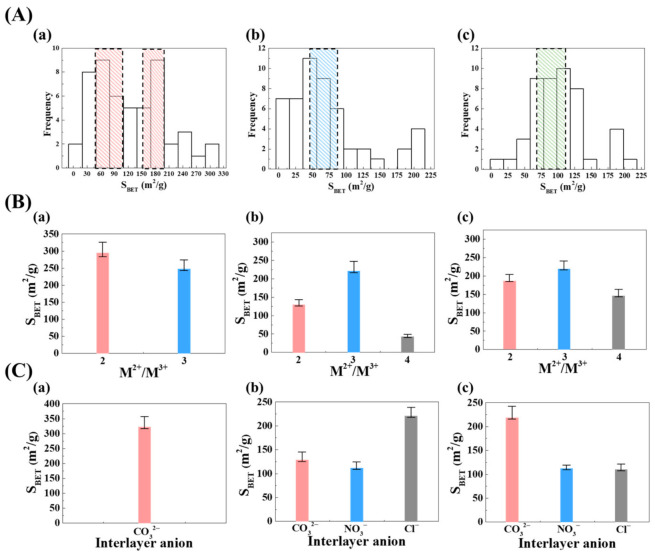
(**A**) Histogram for the specific surface are values of depending on metal composition. Dotted box stands for the most frequently reported value domain. (**B**) Average S_BET_ and standard deviation of three MMOs depending on metal ratio, (**C**) Average S_BET_ and standard deviation of three MMOs depending on interlayer anion. (**a**) MgAl-MMO, (**b**) ZnAl-MMO, and (**c**) MgFe-MMO. The graphs in (**B**,**C**) were prepared with selected values from the literature used in (**A**).

The effect of metal species on S_BET_ was analyzed first and displayed in Figure 1A. The (average value) ± (standard deviation) of S_BET_ was calculated 141.8 ± 76.6 m^2^/g, 81.8 ± 56.7 m^2^/g, and 117.5 ± 45.9 m^2^/g, for MgAl-, ZnAl- and MgFe-MMO, respectively, suggesting that the specific surface area values were broadly distributed and that there is no statistical difference among composition. However, in-depth analyses suggested that most of the values were located in a certain range: two domains 50–95 m^2^/g and 165–205 m^2^/g for MgAl-MMO (22 out of 50 examples, dotted box in Figure 1(Aa)), 45–85 m^2^/g for ZnAl-MMO (23 out of 50 examples, dotted box in Figure 1(Ab)), and 65–110 m^2^/g for MgFe-MMO (16 out of 20 examples, dotted box in Figure 1(Ac)). The statistics suggests that metal composition of initial LDH might influence the specific surface area of MMO.

Metal ratio between divalent and trivalent did not critically affect the S_BET_ of MMO in MgAl and MgFe composition (Figure 1B). However, the S_BET_ of ZnAl-MMO was fairly dependent on the metal ratio, showing the largest value in 3/1 ratio. It is not clear at this stage how metal ratio affect the specific surface area, it may be related to the role of trivalent ion in MMO, connecting metal oxide domains in interstratified manners. In order to fulfill the bridging role, the trivalent ion should be migrated to tetrahedral site from octahedral site. The degree of migration in trivalent ion seems to be dependent on metal ratio in ZnAl-LDH. The 3/1 ratio, which is the most stabilized ratio in hydrotalcite-like structure, seemed to facilitate Al migration to tetrahedral site to support porous structure in MMO.

**Figure 2:**
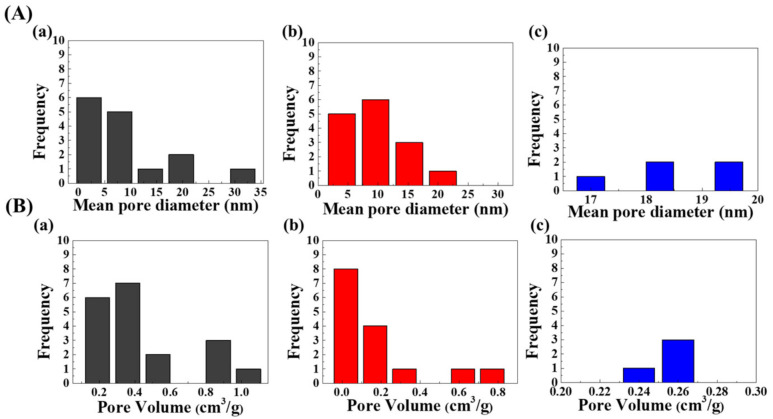
Histograms for the (**A**) mean pore diameter and (**B**) pore volume depending on metal composition. (**a**) MgAl-MMO, (**b**) ZnAl-MMO, and (**c**) MgFe-MMO.

According to our research, the interlayer anion was also determined to affect specific surface area of MMO. In fact, most of MgAl-MMO were prepared from carbonated-LDH, and thus we could only compare the anionic effect in ZnAl and MgFe-MMO. As shown in Figure 1C, ZnAl-MMO showed the largest S_BET_ values with chloride-LDH, while MgFe-MMO resulted in high S_BET_ from carbonated-LDH. It is obvious that nitrated-LDH was not advantageous in producing high specific surface area. When carbonated or chloride-LDHs are calcined in carbon dioxide or hydrochloric acid gas, respectively, it evolves, resulting in pores. However, nitrated-LDH is considered to produce various NO_x_ species upon calcination. This unidentified gasification of interlayer anion is attributed to the less development of specific surface area. In addition to the specific surface area, pore size or pore volumes depending on metal composition were also analyzed. As shown in the histogram of Figure 2A, the mean pore diameter of all the MMOs lay in the mesopores region. The MgAl-MMO showed mean pore dimeter values smaller than those of ZnAl or MgFe-MMO. The average value of mean pore diameter calculated from the data in Figure 2A were 10.93 nm 12.88 nm, and 18.75 nm, respectively, for MgAl, ZnAl, and MgFe-MMO. This clearly showed that MgAl-composition is the most favorable in developing small pores. The pore volume analysis also showed advantages in MgAl-composition. As shown in Figure 2B, pore volume tended to be higher in MgAl-MMO. The average pore volume were 0.529 cm^3^/g, 0.214 cm^3^/g, and 0.278 cm^3^/g, respectively, for MgAl, ZnAl, and MgFe-MMO. This tendency could be explained by not only the different surface energy between MgO and ZnO but also the tetrahedral fitting of Al^3+^ compared with Fe^3+^. Due to the relatively low surface energy, MgO tended to preserve smaller crystallite than ZnO; migration of Al from octahedral to tetrahedral site was more facilitated than that of Fe, resulting in a hierarchically interstratified structure.

Although it cannot be explained by a single rule, the characteristic of initial LDH, synthetic and calcination condition would be related to the property of final MMO. In the successive section, we introduce several examples of MgAl-MMO, ZnAl-MMO and MgFe-MMO to suggest specific condition to control specific surface area.

- MgAl-MMO

LDH consists of Mg and Al is known as mineral hydrotalcite which has general formula of Mg_6_Al_2_(OH)_16_(CO_3_)∙4H_2_O. Due to the abundance of Mg^2+^, Al^3+^, and carbonate in the earth’s crust, hydrotalcite usually occurs in nature; furthermore, it is relatively simple and economic to prepare in a laboratory scale. Thermal treatment of hydrotalcite (MgAl-LDH) under mild temperature from 300 to 600 °C results in the formation of small domains of MgO (periclase, JCPDS No. 14-0191) with connecting Al^3+^ (MgAl-MMO) [54,55]. This phenomenon is one of the major reasons for the development of porosity. Above 600 °C, there evolves thermodynamically stable spinel phase (MgAl_2_O_4_, JCPDS No. 77-1913), hindering the intended structure of MMO with high porosity [56,57].

**Figure 3:**
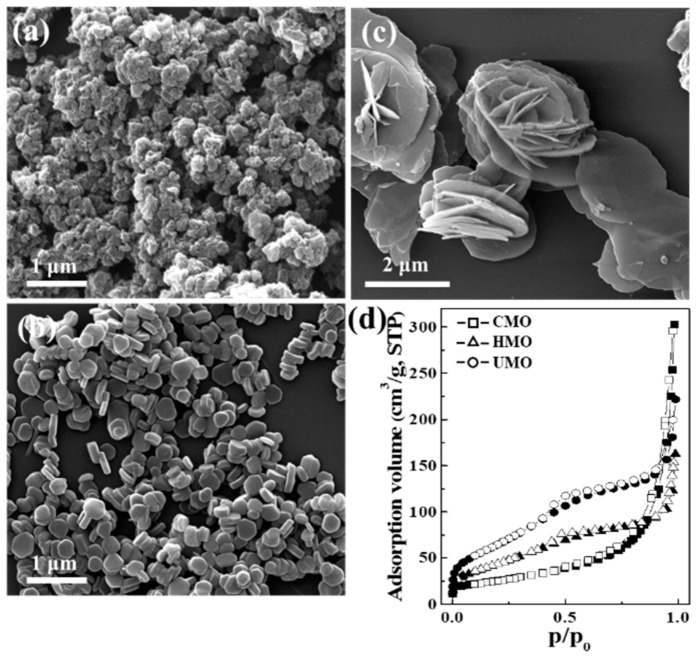
Scanning electron microscopy (SEM) images of (**a**) CHO, (**b**) HMO, and (**c**) UMO. CMO, HMO, and UMO are results of calcination of LDH that synthesized by coprecipitation, hydrothermal, and urea-hydrolysis, and (**d**) N_2_ adsorption-desorption isotherms for CMO, HMO, and UMO (adapted from [58] with the permission from Elsevier, 2020).

We recently found that the specific surface area values of MgAl-MMO were dependent on the synthetic method of pristine LDH [58]. Three kinds of MMOs were prepared from pristine LDHs synthesized by coprecipitation only, hydrothermal treatment after coprecipitation, and urea hydrolysis-based coprecipitation; each MMO was designated as CMO, HMO, and UMO, respectively. According to SEM measurement (FEI QUANTA 250 FEG, Hillsboro, OR, USA), it was revealed that the three MMOs had comparable particle size with its pristine: 49.45 ± 12.85 nm, 224.86 ± 18.91 nm, and 1999.68 ± 255.48 nm for CMO, HMO, and UMO, respectively (Figure 3a–c).

Generally, small particle size resulted in more adsorption and larger specific surface area; however, the S_BET_ of each MMO was determined to have reverse relationship: 91.3 m^2^/g for CMO, 168 m^2^/g for HMO, and 250 m^2^/g for UMO (obtained by BELSORP-mini Ⅱ, Microtrac BEL, Inc., Tokyo, Japan). Furthermore, micropore contribution was more clearly shown in larger particle such as UMO. These results clearly suggest that one MMO particle had small MgO domains and that those domains were more regularly arranged resulting in large intraparticle space and high S_BET_ (Figure 3d).

**Figure 4:**
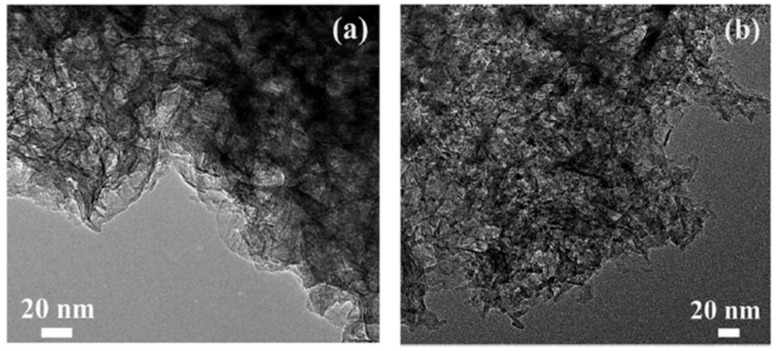
Transmission electron microscopy (TEM, JEM-2100F electron microscope, JEOL Ltd., Tokyo, Japan) images of (**a**) MgAl-LDH and (**b**) MgAl-MMO (adapted from [59] with permission with Royal Society of Chemistry, 2016).

The MgAl-MMO with relatively high specific surface area (>250 m^2^/g) could be obtained by applying particular methods in addition to coprecipitation. Yan et al. [59] achieved an S_BET_ value of 316 m^2^/g for MgAl-MMO by preparing pristine LDH under water-in-oil microemulsion condition. Aqueous metal cations (Mg^2+^ and Al^3+^) were titrated with ammonia water inside a microemulsion developed by polyethylene glycol and cyclohexane. The pristine LDH obtained by this method showed assembly of small and thin particles compared with LDHs prepared by coprecipitation alone (Figure 4a).

**Figure 5:**
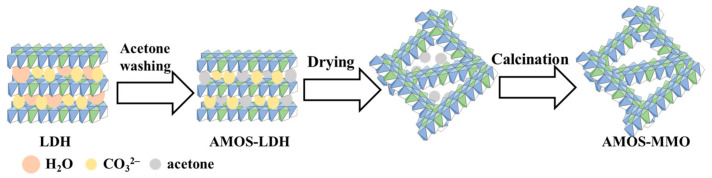
The schematic diagram of synthesis of AMOS-LDH and AMOS-MMO (adapted [61] with permission from Elsevier, 2019).

After calcination, the building block particles of agglomerates became to have thinner and smaller nanosheets morphology and the nanosheets were interconnected to each other to form porous structure (Figure 4b) [59]. In the introduction, we hypothesized that porosity of MMO is originated from both intraparticle space among MgO domains and interparticle cavity. Careful prevention of aggregation among particles by microemulsion method seemed to maximize interparticle cavity to enhance S_BET_. Another example to obtain high specific surface area in MgAl-MMO is utilizing aqueous miscible organic solvent (AMOS) treatment. This method has been frequently utilized to enhance specific surface area of pristine itself [60]. Zhu et al. [61] prepared LDH through conventional coprecipitation and washed the slurry with acetone (purchased from Sigma-Aldrich Inc., St. Louis, MO, USA). Upon rinsing with acetone, the interlayer water was removed and the interaction between the LDH sheets was weakened, resulting in the exfoliation. During vacuum drying, there occurred reassembly of LDH nanolayers, giving rise to a flower-like structure in which thinner layers were inter-connected (Figure 5).

The AMOS-LDH itself had higher specific surface area value (299 m^2^/g) than conventionally prepared LDH (78 m^2^/g). As expected, the AMOS-MMO also had much higher S_BET_ (325 m^2^/g) than MMO without acetone treatment (256 m^2^/g) (obtained by TriStar Ⅱ Plus, Micromeritics Instruments Corporation, Norcross, GA, USA). The high S_BET_ value in this example is also interpreted as the enhancement in interparticle cavity. The hierarchical layer-stacking structure of AMOS-LDH could be robustly maintained during calcination preserving the particle-particle distance, finally maximizing the adsorption site.

- ZnAl-MMO

Similar to MgAl-LDH, thermal treatment on ZnAl-LDH caused the partial collapse in lamellar structure through degassing. Calcination at moderate temperature from 300 °C to 600 °C resulted in divalent metal oxide (ZnO) [62,63,64]. Different from MgAl-MMO, however, the ZnAl-MMO tends to have lower specific surface area (Figure 1(Ab)). The tendency could be interpreted in terms of different chemical nature between ZnO and MgO. According to the literature, the solid-vapor interface energy was 1.07 J/m^2^ and 2.55 J/m^2^ for MgO and ZnO, respectively [65]. This information suggested that the MgO nanocrystallites can be more stably separated than ZnO, implying aggregation of ZnO domains. The XRD results from many literatures also showed higher crystallinity of ZnAl-MMO than MgAl-MMO, reflecting the different interface behavior. Based on Gidado et al.’s report [66], we could calculate the crystallinity of both MMOs which were calcined at the same condition. The crystallite size calculated by Scherrer’s equation by ourselves was 3.83 Å for ZnAl-MMO (along (100) plane) and 2.88 Å for MgAl-MMO (along (200) plane), strongly suggesting the facilitated crystal growth of ZnO compared with MgO. According to the hypothesis we suggested in the introduction, the intraparticle space would be smaller in ZnAl-MMO than in MgAl-MMO and the final specific surface area tends to be small in ZnAl-MMO.

As mentioned above, most of the S_BET_ values of ZnAl-MMO ranged between 45 m^2^/g to 85 m^2^/g, exhibiting relatively narrow distribution compared with MgAl-MMO. Huang et al. [67] reported that calcination of hydrothermally prepared ZnAl-LDH resulted in S_BET_ of 32.9 m^2^/g, which was a fairly small value. Zhang et al. [68] synthesized ZnAl-LDH through urea hydrolysis method and calcined it and obtained specific surface area values 70.94 m^2^/g, 62.91 m^2^/g, and 60.41 m^2^/g at a calcination temperature of 500, 600, and 700 °C, respectively (obtained by Nova Quantachrome 4000e, Quantachrome Instruments, Boyton Beach, FL, USA). The authors interpreted that the decrease in S_BET_ with respect to temperature was attributed to the formation of stoichiometric spinel phase with increasing temperature. Starukh and Levytska [69] coprecipitated ZnAl-LDH in various Zn/Al ratio (2/1, 3/1, and 4/1) and calcined them at various temperature from 450 °C to 600 °C. The S_BET_ of their ZnAl-MMO distributed between 45 m^2^/g and 94 m^2^/g (obtained by Nova Quantachrome 2200e, Quantachrome Instruments, Boyton Beach, FL, USA), which corresponded to the major distribution range displayed in Figure 1(Ab).

**Figure 6:**
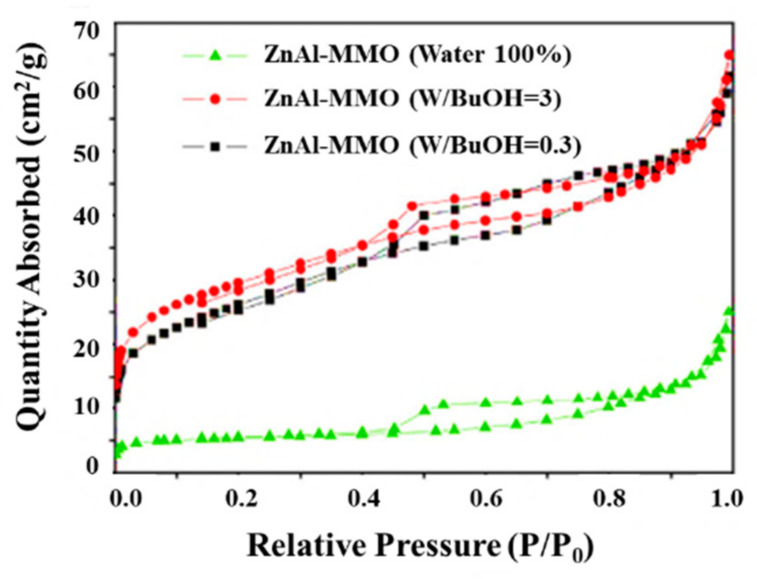
N_2_ adsorption-desorption isotherms of ZnAl-MMO with water, water/BuOH = 0.3 and 3 (adapted from [70] with permission from Elsevier, 2017).

Meanwhile, researchers attempted to obtain high specific surface area in ZnAl-MMO by adopting unique solvents during the synthesis process or introducing interlayer anions other than carbonate. Huang et al. [70] synthesized ZnAl-LDH under water/n-BuOH mixed solvent condition where the water/n-BuOH ratio was controlled 0.3 and 3, respectively. As shown in the N_2_ adsorption-desorption isotherms (ASAP 2460 instruments, Micromeritics Instruments Corporation, Norcross, GA, USA), the ZnAl-LDH prepared under mixed solvent resulted in MMOs with higher nitrogen adsorption (Figure 6).

- MgFe-MMO

The MgFe-MMO originated from MgFe-LDH was often reported as adsorbents for organic dyes. Different from Al-based MMOs, it is free from Al-related disease such as Alzheimer syndrome [71]. In addition, it is possible to provide iron-based MMOs with magnetic property, which facilitates recovery of adsorbents after scavenging pollutant [72,73]. Similar to the other composition, MgFe-LDH undergoes thermal decomposition at 400–600 °C to produce porous MMO and transform to mixture of MgO and MgFe_2_O_4_ spinel phase (JCPDS No. 88-1943) at higher temperature. Therefore, most of the calcination process for MgFe-MMO was reported in the temperature range aforementioned.

For instance, Parida et al. [71] prepared MgFe-MMO by using coprecipitated MgFe-LDH as precursor and by calcining it at 500 °C for 7 h. The specific surface area was 168 m^2^/g (obtained by Qunatasorb-evo, Quantachrome Instruments, Boyton Beach, FL, USA) and exhibited high removal efficiency of selenite from aqueous solution with adsorption capacity 40 mg/g. Similarly, Yu et al. [74] utilized LDH-originated MgFe-MMO to remove aqueous methyl orange (MO) (purchased from Shanghai Chemical Industry Company, Shanghai, China). They precipitated MgFe-LDH with different Mg/Fe molar ratio, treated precipitates under hydrothermal condition (120 °C, 24 h), and calcined LDH at 500 °C for 6 h. Due to the highly crystalline nature of hydrothermally prepared LDH, the S_BET_ of prepared MMO lay in the low value range 38.2–48.7 m^2^/g (obtained from Micromeritics ASAP 2020, Micromeritics Instruments Corporation, Norcross, GA, USA). However, due the well-developed pore structure, the material showed fairly high adsorption capacity of 194.9 mg-MO/g-MMO. Another example of MgFe-MMO was reported to remove aqueous phosphate (purchased from Fengchuan Chemical Co. Ltd., Tianjin, China) [75]. Coprecipitated MgFe-LDH was calcined at different temperature (200–600 °C) for 3 h. The S_BET_ of MMO was the largest at calcination temperature 300 °C showing 85.6 m^2^/g, while the increasing temperature resulted in the decrease of specific surface area.

#### 2.2.2. Temperature Effect on LDH

As previously mentioned, the calcination of LDH gradually releases hydroxyls and interlayer anion, accompanying collapse of the layered structure. At moderate calcination temperature, 300–600 °C, the LDH undergoes thermal decomposition to metal oxide. Below 300 °C the layered structures still remained with relatively low crystallinity. From 300 °C, there appears metal oxide phase due to the dehydroxylation in the framework. Within the increasing in calcination temperature (~600 °C), the characteristic LDH phase finally disappeared, indicating the destruction of the lamellar structure, which was related to the gasification of interlayer anion. Above 600 °C, the generation of spinel phase and disappearance of MMO phase occurred simultaneously and the spinel phase become more crystalline with increasing temperature, negatively influencing the S_BET_ value.

**Figure 7:**
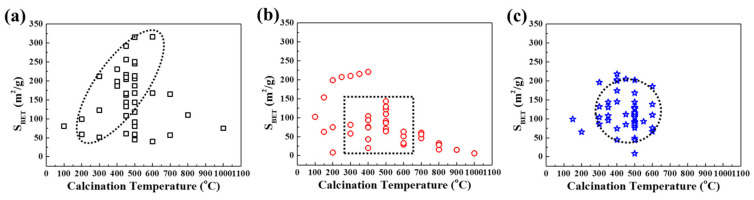
S_BET_ distribution patterns by calcination temperature of (**a**) MgAl-MMO, (**b**) ZnAl-MMO, and (**c**) MgFe-MMO. Each dotted region indicates the most frequently observed S_BET_ values.

Literature research on the S_BET_ of MMO gave us an inspiration that calcination temperature within 300–600 °C played an important role in controlling the specific surface area. The S_BET_ distribution pattern of MgAl-MMO, ZnAl-MMO, and MgFe-MMO with respect to calcination temperature is displayed in Figure 7.

The MgAl-MMOs exhibited a wide S_BET_ distribution from 50 m^2^/g to 325 m^2^/g in the calcination temperatures range between 300–600 °C (dotted ellipsoid in Figure 7a). The tilted ellipsoid showed increasing S_BET_ tendency with respect to calcination temperature. This pattern was clearly demonstrated in Zhang et al.’s work [76]. They prepared MgAl-LDH by conventional coprecipitation method and thermally treated it at various temperatures from 100–600 °C. Calcination temperatures at 100 °C and 200 °C resulted in the remaining of LDH phase with low specific surface area, 80.7 m^2^/g and 98.9 m^2^/g. From 300–600 °C, the MMO phase was dominant and the S_BET_ value increased upon temperature: 122.2 m^2^/g, 198.4 m^2^/g, and 245.2 m^2^/g at 300 °C, 400 °C, and 500 °C, respectively. Over 600 °C, due to the gradually formed spinel phase, the S_BET_ decreased to 168.2 m^2^/g (obtained by NOVA 1000e, Quantachrome Instruments, Boyton Beach, FL, USA). The propensity could be addressed by the thermal behavior of MgAl-LDH. As generally depicted in the thermogravimetric and calorimetric analyses (obtained by Shimadzu DT-40, Shimadzu Scientific Instruments, Kyoto, Japan), MgAl-LDH undergoes dehydroxylation and degassing of interlayer anion mainly in the temperature 380–430 °C (peak maximum); weight loss was continuously observed until 600 °C [77,78]. This stands for that the intraparticle pore (Scheme 1) could be enlarged until 600 °C with increasing S_BET_ value.

Similarly, ZnAl-MMO presented increasing S_BET_ up to 500 °C and decreasing afterward, although most of the S_BET_ values were observed between 30–150 m^2^/g (dotted rectangle in Figure 7b). The thermogravimetric and calorimetric study (obtained by NETZSCH STA 409 CD, NETZSCH Holding, Selb, Germany; Derivatograph Q-1599 D, MOM, Hungary) showed that the weight loss of ZnAl-LDH continuously occurred until 300 °C [79,80], suggesting that the increasing intraparticle pore depending on temperature. However, Zhang and Li’s study showed the decreasing S_BET_ value at 600 °C [81]. This phenomenon could be explained by the high interface energy of ZnO [65], resulting in particle growth during calcination.

The specific surface area of MgFe-MMO did not show temperature-dependency in the temperature from 300–600 °C, showing most of the S_BET_ values in the region between 30–150 m^2^/g with fairly independent distribution regardless of temperature (dotted circle in Figure 7c). It is not clear why MgFe-MMO tend to have smaller S_BET_; however, one possible explanation is the role of trivalent cation in MgFe-MMO. It was explained in the introduction that Al^3+^ in the octahedral site of LDH partially migrates to tetrahedral site of MMO, which interconnects M(II)O domains in MMO, producing intraparticle pore. On the other hand, Fe^3+^ is not appropriate to take the inter-connection role in tetrahedral site due to the following reason. The ionic radius of Fe^3+^, Al^3+^, and O^2−^ were 70 pm, 53 pm, and 120 pm, respectively. Considering the ionic radius ratio (0.42 for Fe^3+^/O^2−^ and 0.37 for Al^3+^/O^2−^), and Fe^3+^ was more stable in octahedral interstices than in tetrahedral hole [82]. Due to the size discrepancy, the migration of Fe^3+^ from octahedral to tetrahedral hole would be restricted, resulting in the less development of porous structure.

### 2.3. Effect of Pore Developoing Agent

**Figure 8:**
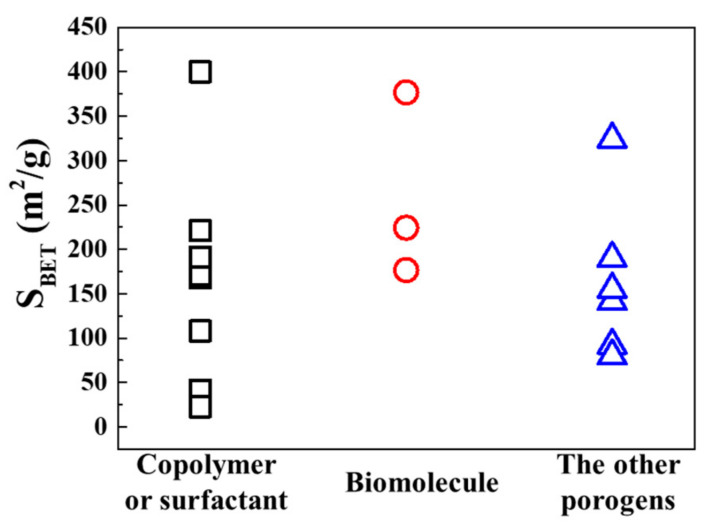
The reported S_BET_ value of porous MMO prepared by the porogen-LDH. Porogen: micelle forming copolymer or surfactants (open square), biomolecules (open circle), the other porogens (open triangle).

The specific surface area of MMO can be finely tuned by utilizing nanometer-sized pore developing agent, porogen, in LDH synthesis. Thermal treatment decomposes porogen in the remaining void space. Size and arrangement of porogen influences dimension and homogeneity of pore in MMO, influencing the specific surface area (Scheme 1). Literature research revealed that the S_BET_ value had wide distribution (Figure 8); however, the regularity in porous structure of LDH-originated MMO was better controlled with the help of porogen. Exact S_BET_ values depending on the type of porogen was summarized in Table 2 with citation number.

- Micelle-Forming Copolymer and Surfactant

One of the most widely utilized porogens is pluronic block copolymer such as F127 and P123, which have been well-demonstrated in the synthesis of mesoporous silica [83,84,85,86]. For example, Wang et al. [87] obtained mesoporous MgAl-MMOs utilizing different concentration of P123 solution (purchased from Sigma-Aldrich Inc., St. Louis, MO, USA) during coprecipitation process. Although their maximum S_BET_ value was not very high (108.1 m^2^/g, obtained from Micromeritics ASAP 2010, Micromeritics Instruments Corporation, Norcross, GA, USA), the MMO showed fairly homogeneous nanopores which could be further utilized as reservoir for drug. Similar concept of research was also reported with NiAl-MMO which was prepared from F127 (purchased from Sigma-Aldrich Inc., St. Louis, MO, USA) templated LDH. The paper reported that F127 acted as a template to produce homogenous mesopores with fairly high S_BET_ of 184 m^2^/g in MMO and that the homogeneous pore was beneficial in catalytic activity toward Knoevenagel condensation reaction [88]. Kuroda et al. [89] applied F127 (purchased from Sigma-Aldrich Inc., St. Louis, MO, USA) to produce MMO with high S_BET_ and homogeneous pore (obtained by Quantachrome Autosorb iQ_2_, Quantachrome Instruments, Boyton Beach, FL, USA). Different from the other approaches utilizing pluronic incorporation during coprecipitation, they homogeneously combined monodispersed MgAl-LDH with F127. As a result, they could achieve very high specific surface area of 400 m^2^/g. We recently showed that the type of solvent controlled molecular aggregation of pluronic polymer during LDH synthesis, strongly influencing the specific surface area of MMO [90]. In the study, P123-incorporated LDH was coprecipitated on two types of solvent, ethanol and water. It was shown that the P123 aggregated strongly under ethanol condition, while the nanometer-sized micelles were obtained in water system. The specific surface area values of MMOs that were obtained from LDHs synthesized in ethanol and water were 179 m^2^/g and 221 m^2^/g, respectively, suggesting that the molecular arrangement of porogen affected the S_BET_ of MMO (obtained by BELSORP-mini Ⅱ, Microtrac BEL, Inc., Tokyo, Japan). Furthermore, thus obtained MMO with high surface area exhibited dramatically high adsorption capacity of 3470 mg/g toward Congo Red dye (purchased from Sigma-Aldrich Inc., St. Louis, MO, USA).

Meanwhile, researchers also reported that micelle-forming molecular surfactant could be utilized as porogen for MMO production. He et al. selected dioctyl sulfosuccinate sodium (purchased from Sigma-Aldrich Inc., St. Louis, MO, USA). as a porogen for MgAl-MMO [91]. As the concentration of surfactant increased, the specific surface area of MMO increased from 41.43 m^2^/g to 191.36 m^2^/g (obtained from Micromeritics Rise 1030, Micromeritics Instruments Corporation, Norcross, GA, USA), which was much higher than the S_BET_ of MMO obtained without porogen (15.35 m^2^/g). Another research reported that cationic surfactant, cetyltrimethylammonium bromide, can be utilized to prepare mesoporous ZnAl-MMO [92]. Although the S_BET_ (22.57 m^2^/g) of the prepared ZnAl-MMO was not high (obtained from Micromeritics ASAP 2020, Micromeritics Instruments Corporation, Norcross, GA, USA), they suggested the potential utility of micelle-forming surfactant in the preparation of homogenous mesopores in MMO.

- Biomolecules

**Figure 9:**
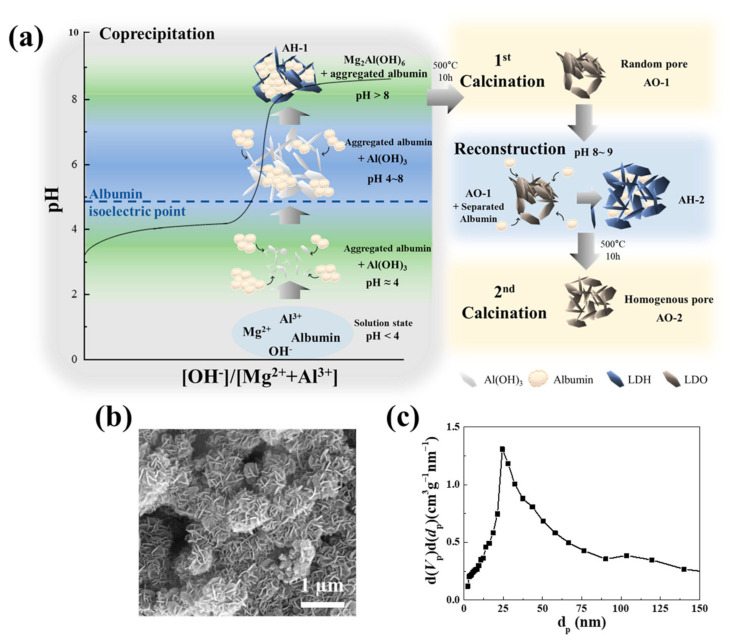
(**a**) Schematic diagram to produce MMOs from either coprecipitated albumin-LDH or reconstructed albumin-LDH. (**b**) SEM image (obtained by FEI QUANTA 250 FEG, Hillsboro, OR, USA) and (**c**) pore size distribution of MMO obtained from reconstructed albumin-LDH (obtained by BELSORP-mini Ⅱ, Microtrac BEL, Inc., Japan) (adapted with the permission from Jung et al. [48]. Copyright MDPI).

The above-mentioned porogen facilitated formation of self-assembled micelles, resulting in the development of pores after removal. Similar strategy is possible with intrinsically nano-sized biomolecules. We recently found that calcination of albumin-templated LDH produced MgAl-MMO with fairly high specific surface area. In this study, albumin (purchased from Sigma-Aldrich Inc., St. Louis, MO, USA) was added during two different LDH synthesis routes, coprecipitation and reconstruction, respectively (Figure 9a) [48].

The MMO obtained from reconstructed albumin-LDH had higher S_BET_ (224.3 g/m^2^) than MMO from coprecipitated albumin-LDH (S_BET_ = 176.6 m^2^/g). This was attributed to the different degree of aggregation of albumin depending on synthetic condition; reconstruction reaction preserves constant pH value, resulting in the homogeneous distribution of albumin as porogen. Thus, the reconstructed MMO contained well-developed inter-particle pore (Figure 9b) in addition to intra-particle pore resulting in narrow pore size distribution (Figure 9c). Xu et al. [93] utilized sodium alginate (purchased from Sinopharm Chemical Reagent Co., Ltd., Shanghai, China) as a porogen for MgAl-MMO. They coprecipitated MgAl-LDH in the presence of sodium alginate solution. The alginate-templated LDH resulted in MMO with fairly high S_BET_ of 376.8 m^2^/g. Biological organism with hierarchical structure such as rape pollen was also be utilized as porogen. Duan et al. [94] precipitated ZnAlCe-LDH in the presence of rape pollen and calcined it at 550 °C. The MMO was obtained in a large particle morphology with hierarchically connected pores. They showed that the porous MMO had excellent activity as heterogenous catalysis.

- The other porogens

Self-templating synthesis of MMO could be an alternative method to evolve mesopores and enhance specific surface area of MMO. Chen et al. [95]. transformed ZIF-67 to metal-organic frameworks into 3D hollow structure of LDH. Upon successive etching and calcination, they could obtain hierarchical nanocage of NiCO_2_O_4_/NiO composition. The MMO showed moderate S_BET_ value of 141.6 m^2^/g (obtained by AS-1-C TCD, Quantachrome Instruments, Boyton Beach, FL, USA) and excellent catalytic activity. 

Glucose template was another approach to generate mesoporous MMO. Li et al. [96] proposed NiAl-LDH/carbon composite as a precursor for porous MMO. First, LDH was prepared with glucose, and then hydrothermally treated at 150 °C, at which condition the glucose underwent carbonization. The removal of carbon by calcination resulted in MMO with high specific surface area and interconnected mesopores (obtained by Quantachrome Autosorb-1C-VP, Quantachrome Instruments, Boyton Beach, FL, USA). Similarly, Feng et al. [97]. developed spinel type MgAl-MMO by coprecipitation and hydrothermal process in the presence of glucose. The MgAl_2_O_4_ spinel exhibited high specific surface area of 324 m^2^/g. The organic glucose contributed to the evolution of mesopores and high specific surface area (obtained by Micromeritics TriStar 3000, Micromeritics Instruments Corporation, Norcross, GA, USA).

## 3. Conclusions

Application and utility of MMO is increasing in various fields such as catalysts, electrodes, adsorbents, construction, environment, etc. due to their diverse composition and high specific surface area. One of the recent approaches to prepare MMO is calcining LDH; thermal treatment on LDH gives rise to dehydration, simultaneous dehydroxylation, and degassing of anion, and development of the spinel phase depending on temperature. According to the literature survey, calcination at moderate temperature 300–600 °C resulted in pure MMO with fairly high specific surface area. Calcination at lower or higher temperature resulted in the mixture of (hydrotalcite + MMO) or (spinel + MMO) to decrease the specific surface area. It is expected that there are two reasons of S_BET_ increase at moderate temperature: (i) formation of intraparticle pore coming from the degassing and (ii) interparticle space which is strongly governed by the interaction of particles.

As for the metal composition effect on S_BET_, MgAl-MMO generally tended to show relatively large specific surface than ZnAl-MMO (45–85 m^2^/g), while MgFe-MMO had wide range of S_BET_. This may be highly related to the surface energy of divalent metal oxide (MgO or ZnO) and site preference of Al^3+^ and Fe^3+^. In order to achieve very high S_BET_, specific synthetic process such as AMOST, water-n-BuOH solvent, water-in-oil microemulsion method were applied; in this way, thinner and small pristine LDH particles could be prepare and the inter-particle space would be well-preserved to enhance high specific surface area. Calcination temperature was determined to be related with specific surface area, showing generally increasing S_BET_ upon temperature. In addition, the utility of pore-developing agent was also adapted in LDH-originated MMO. Although the S_BET_ value did not dramatically increase in the presence of porogen, it could develop homogeneous porous structure, which is advantageous in adsorbent or catalyst.

## Figures and Tables

**Table 1:** Specific surface area values of MMOs depending on metal composition, interlayer anion, and calcination temperature.

Metal Composition	Interlayer Anion	Temperature (°C)	S_BET_ (m^2^/g)	Ref.
MgAl	CO_3_^2−^	450 °C	204 m^2^/g	[4]
		450 °C	75–143 m^2^/g	[8]
		450 °C	108–163 m^2^/g	[8]
		450 °C	216–292 m^2^/g	[9]
		450 °C	59 m^2^/g	[12]
		450 °C	209 m^2^/g	[14]
		450 °C	60–133 m^2^/g	[16]
		450 °C	32.9 m^2^/g	[17]
		500 °C	180–210 m^2^/g	[1]
		500 °C	117.6 m^2^/g	[7]
		500 °C	91–250 m^2^/g	[15]
		700 °C	165.1 m^2^/g	[11]
		750 °C	80.4 m^2^/g	[13]
		300–600 °C	210–320 m^2^/g	[2]
		200–1000 °C	55–150 m^2^/g	[3]
		400–600 °C	38–187 m^2^/g	[5]
		300–700 °C	44–58 m^2^/g	[6]
		200–600 °C	80–246 m^2^/g	[10]
ZnAl	CO_3_^2−^	370 °C	80 m^2^/g	[30]
		400 °C	20–104 m^2^/g	[25]
		450 °C	32.9 m^2^/g	[29]
		500 °C	122 m^2^/g	[23]
		500 °C	129 m^2^/g	[26]
		500 °C	28–86 m^2^/g	[27]
		500 °C	143.3 m^2^/g	[28]
		500 °C	109.5 m^2^/g	[7]
		300–600 °C	51–91 m^2^/g	[24]
		200–1000 °C	6–43 m^2^/g	[18]
	NO_3_^−^	750 °C	22.5 m^2^/g	[20]
		150–600 °C	29–82 m^2^/g	[31]
		300–700 °C	15–46 m^2^/g	[19]
		500–700 °C	60–71 m^2^/g	[21]
	Cl^−^	50–400 °C	102–221 m^2^/g	[22]
MgFe	CO_3_^2−^	350 °C	108–144 m^2^/g	[45]
		350–550 °C	92–96 m^2^/g	[44]
		400 °C	74 m^2^/g	[48]
		450 °C	205 m^2^/g	[46]
		500 °C	168 m^2^/g	[32]
		500 °C	44.3 m^2^/g	[35]
		500 °C	81–127 m^2^/g	[37]
		500 °C	86–128 m^2^/g	[42]
		600 °C	185 m^2^/g	[43]
		600 °C	110 m^2^/g	[47]
		150–600 °C	99–202 m^2^/g	[38]
		300–500 °C	104–196 m^2^/g	[39]
		400–500 °C	116–218 m^2^/g	[40]
		200–600 °C	8–49 m^2^/g	[33]
		450–550 °C	92–112 m^2^/g	[41]
	NO_3_^−^	300–700 °C	65–86 m^2^/g	[34]
		500–600 °C	78–110 m^2^/g	[36]
	Cl^−^	500–600 °C	75–110 m^2^/g	[46]

**Table 2:** Specific surface area values of MMOs depending on the types of porogen.

Porogen		Metal Composition	S_BET_ (m^2^/g)	Ref.
Micelle forming copolymer or surfactants	P123	MgAl	13–180 m^2^/g	[87]
			179–221 m^2^/g	[90]
	F127	NiAl	97–184 m^2^/g	[88]
		MgAl	239–400 m^2^/g	[89]
	Dioctyl sulfosuccinate	Mg	41–192 m^2^/g	[91]
	cetyltrimethylammonium bromide	ZnAl	9–31 m^2^/g	[92]
Biomolecule	Albumin	MgAl	176–225 m^2^/g	[48]
	Sodium Alginate	MgAl	123–377 m^2^/g	[93]
	Rape pollen	ZnAlCe	97–113 m^2^/g	[94]
The others	MOF	CoNi	64–127 m^2^/g	[95]
	Glucose	NiAl	144–288 m^2^/g	[96]
		MgAl	151–323 m^2^/g	[97]

## Data Availability

No new data were created or analyzed in this study. Data sharing is not applicable to this article.
